# Dietary resveratrol improves the flesh quality of Siberian sturgeon *(Acipenser baerii)* by enhancing myofiber growth, nutrient accumulation and antioxidant capacity

**DOI:** 10.1186/s12864-024-10436-6

**Published:** 2024-05-24

**Authors:** Shiyong Yang, Jiajin Zhang, Zihan Xu, Wuyuntana Shao, Xiaojian Pang, Datian Li, Xiaoli Huang, Wei Luo, Zongjun Du, Yunkun Li, Jiayun Wu, Xiaogang Du

**Affiliations:** 1 Department of Aquaculture, College of Animal Science & Technology, Sichuan Agricultural University, Chengdu, 611130 China; 2 Department of Engineering and Applied Biology, College of Life Science, Sichuan Agricultural University, Ya’an, 625014 China

**Keywords:** Resveratrol, Siberian sturgeon, Flesh quality, Antioxidative ability, Muscle, Transcriptome

## Abstract

**Background:**

In aquaculture, sturgeons are generally maintained in the confined spaces, which not only hinders sturgeon movement, but also threatens their flesh quality that seriously concerned by aquaculture industry. As a typical antioxidant, resveratrol can improve the flesh quality of livestock and poultry. However, the mechanism of resveratrol’s effect on the muscle of Siberian sturgeon is still unclear.

**Results:**

In this study, the dietary resveratrol increased the myofiber diameter, the content of the amino acids, antioxidant capacity markers (CAT, LDH and SOD) levels and the expression levels of *mTORC1* and *MYH9* in muscle of Siberian sturgeon. Further transcriptome analysis displayed that ROS production-related pathways (“Oxidative phosphorylation” and “Chemical carcinogenes-reactive oxygen species”) were enriched in KEGG analysis, and the expression levels of genes related to the production of ROS (*COX4, COX6A, ATPeF1A*, etc.) in mitochondria were significantly down-regulated, while the expression levels of genes related to scavenging ROS (*SOD1*) were up-regulated.

**Conclusions:**

In summary, this study reveals that resveratrol may promote the flesh quality of Siberian sturgeon probably by enhancing myofiber growth, nutritional value and the antioxidant capacity of muscle, which has certain reference significance for the development of a new type of feed for Siberian sturgeon.

## Background

Sturgeon, as an ancient species [1, 2], is famous for its precious caviar and delicious meat [2–4]. Among the many sturgeon species, Siberian sturgeon (*Acipenser baerii*) is widely farmed in China because of its rapid growth and stress resistance [5–7]. In recent years, the researches about Siberian sturgeon mainly focused on growth [8], gonadal development [9] and behaviors (such as swimming [10] and feeding [11]). The mostly studied tissues in Siberian sturgeon are brain [12], intestine [13] and liver [14], meanwhile the muscle is less revealed. In particular, the growth of muscle is one of the key factors for muscle quality [15, 16], which is rarely investigated in Siberian sturgeon. However, the flesh quality of aquatic products is drawing greater attention as peoples’ living standards improve [17]. The flesh quality of fish is a complex set of characteristics, including hardness, color, flavor, nutritional value, etc [17, 18].

Noteworthily, deterioration of fish flesh quality is one of the most important issues in aquaculture [19]. One of the reasons for this is that fishes are confined in a limited space for a long time, which will make them lack of exercise, eventually leading to slow down the growth and development of skeletal muscle [20]. Therefore, it is particularly serious to find a suitable way to make up for the loss of flesh quality caused by lack of exercise.

Resveratrol, a nonflavonoid polyphenol originally extracted from grape skins and leaves, has been widely concerned because of its antioxidant, anti-inflammatory, and metabolic regulatory features [21, 22]. The application of resveratrol as feed additive in animal breeding has been studied extensively [23–26]. It has shown that resveratrol has positive effects on growth performance, meat quality, intestinal health, immunity and reproductive performance of swine, poultry and ruminant [22]. More noteworthily, studies have shown that resveratrol beneficially affects the meat quality of duck, pig and beef cattle by improving the color, drip loss and Warner-Bratzler shear force of meat by enhancing the antioxidant capacity of muscle and changing muscle fiber types [27–29]. Thus, we hypothesized that resveratrol could also improve the meat quality of Siberian sturgeon. However, the effect of resveratrol on meat quality of fish has been poorly reported.

The aim of this study was to investigate the effect of resveratrol on the flesh quality of Siberian sturgeon. Resveratrol was supplied into the daily diet and after 45 days, growth performance, nutritional value, antioxidant capacity and transcriptome of the muscle were evaluated. This study revealed the positive effects of resveratrol on flesh quality, which will provide a certain reference basis for the application of resveratrol in aquaculture.

## Materials and methods

### Fish breeding and experimental design

**Fig. 1:**
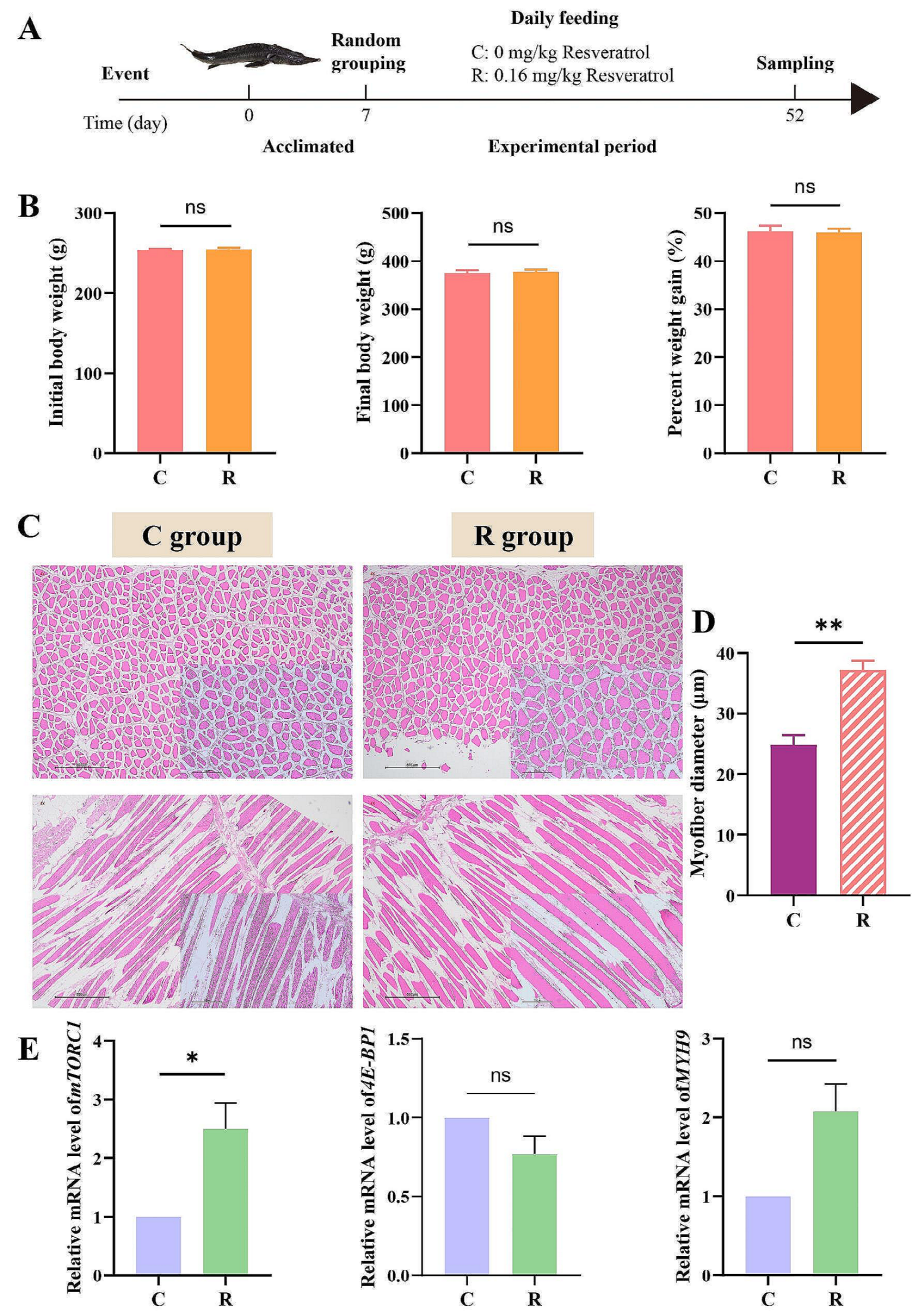
Experimental flowchart, body weight, histological observation and qRT-PCR of Siberian sturgeon. (**A**) Experimental flowchart, sampling at day 52 includes two groups: C (fed with 0 mg/kg resveratrol) and R (fed with 0.16 mg/kg resveratrol). (**B**) The effect of resveratrol on the body weight of Siberian sturgeon. (**C**) Myofiber microstructure of C and R group: Magnification 4× cross sections and longitudinal sections, magnification 10× cross sections and longitudinal sections. (**D**) Myofiber diameter of Siberian sturgeon. (**E**) The relative expression levels of *mTORC1, 4E-BP1* and *MYH9* in muscle of Siberian sturgeon after the resveratrol treatment. Data were shown as mean ± SEM. *p < 0.05, **p < 0.01, ns: no significance

180 Siberian sturgeon (10-month-old, 248.1 ± 5.9 g) were purchased from the fish farm (Tianquan County Chuanze Fishery Co., Ltd., Ya’an, China) and randomly put in circular plastic tanks (1.5 m in diameter and 1 m in height). Fish were raised in the water environment as shown in Table 1, which was stabilized by heating rods, water quality monitors (PTF-001B, XiaMen PanTian BioTech Co., Ltd, China), bottom filter pumps and oxygen pumps, and were fed three times a day (at 8:00 am,14:00 pm, and 20:00 pm, respectively) on 1% the body weight of commercial feed (Haida Group Co., Ltd., Guangdong, China). The main composition of the commercial feed was shown in Table 2. In order to visualize the whole experimental design, we made a concise experimental flow chart (Fig. 1A).

**Table 1:** Parameters of water environment for sturgeon breeding

Water environmental parameters	
Water temperature	16.0 ± 0.5 ℃
Dissolved oxygen	8.0 ± 0.6 mg/L
pH	7.6 ± 0.2
Ammonia nitrogen	≤ 0.01 mg/L
Nitrite	≤ 0.05 mg/L

**Table 2:** The nutrient content of the commercial feed

Nutrients	Content (%)
Crude protein	45.0
Crude fiber	5.0
Crude ash	18.0
Crude lipid	8.0
Total phosphorus	0.8
Lysine	2.3
moisture	10.0

After a week of acclimatization, fish were randomly divided into two groups: C group (fed with a commercial diet) and R group (fed with 0.16 mg/kg of resveratrol, according to our previous study [7]). Each group contained 3 tanks, 30 fish per tank. Resveratrol (purity ≥ 99%) was purchased from Macklin Biochemical Co., Ltd. (Shanghai, China). All animal handling procedures were approved by the Animal Care and Use Committee of Sichuan Agricultural University, following the guidelines of animal experiments of Sichuan Agricultural University under permit number 015-01521300.

### Sampling

After 45 days of feeding, nine fish were randomly selected from each group and anesthetized with 200 mg/L MS-222 (Jinjiang Aquatic Supplies Co., Ltd., Fujian, China). After muscle tissues were sampled, part of them were immediately fixed in 10% neutral formalin buffer for at least 24 h for histological observation, and the rest were placed in liquid nitrogen and then stored at -80℃ for subsequent biochemical assay, amino acids composition analysis, transcriptome sequencing and quantitative real-time polymerase chain reaction (qRT-PCR) validation.

### Histological observation

The fixed muscle tissues were dehydrated, transparentized with xylene, and embedded in paraffin wax. The solidified wax blocks were cut into 4-mm slices and stained with hematoxylin and eosin (H&E) staining. The muscle morphological was observed by using a Nikon TS100 light microscope (Nikon, Tokyo, Japan).

### Biochemical measurements

The activities of catalase (CAT), superoxide dismutase (SOD), lactate dehydrogenase (LDH), total antioxidant capacity (T-AOC) were detected by using commercial kits (Nanjing Jiancheng Bioengineering Institute, Nanjing, China). The concentrations of malondialdehyde (MDA) was measured by using test kit (Nanjing Jiancheng Bioengineering Institute, Nanjing, China). All test operations were performed according to the manufacturer’s recommended protocols.

### Amino acids composition analysis

The amino acid composition was obtained by using an amino acid analyzer. The samples were freeze-dried for 24 h and then grounded to obtain powdered samples, from which hydrolyzed amino acids were extracted. Approximately 20 mg of each sample was suspended in 2 mL of 6 mol/L hydrochloric acid (HCl) and 2 µL of phenol. The ampere tubes were filled with nitrogen for 5 min, sealed, and hydrolyzed in a drying oven for 24 h. The hydrolysates were cooled and transferred to a volumetric flask. 1 mL of the supernatant was centrifuged at 12,000× g for 10 min and dried by a nitrogen blower. Then, 1 mL HCl was added in the dried samples, and the samples were mixed in a shaker. The samples were filtered with a 0.45 μm cellulose filter membrane prior to analysis. Amino acids composition was determined by using an automatic amino acid analyzer (L-8900, Hitachi, Japan).

### RNA extraction, cDNA synthesis, library construction, and Illumina sequencing

Total RNA was extracted from muscle tissue by TRIzol reagent (Invitrogen, USA). Nanodrop2000 (Shanghai, China) was used to detect the concentration and purity of the extracted RNA, and agarose gel electrophoresis was used to detect the RNA integrity. The high-quality RNA sample (OD260/280 = 1.8 ~ 2.2, OD260/230 ≥ 2.0) was used to construct sequencing libraries. Libraries were selected for cDNA target fragments of 200–300 bp on 2% Low Range Ultra Agarose followed by PCR amplified using Phusion DNA polymerase (NEB) for 15 PCR cycles. After quantified by TBS380, paired-end libraries were sequenced by Illumina NovaSeq 6000 platform.

### *De novo* assembly, annotation and analysis

Trinity (https://github.com/trinityrnaseq/trinityrnaseq) was used to assemble the obtained high-quality RNA-seq sequencing data from scratch to generate contig and singleton. Then, TransRate (http://hibberdlab.com/transrate/index.html) and CD-HIT (https://github.com/weizhongli/cdhit) were used to optimize the result of the assembly filter. The assembly results were evaluated by using BUSCO (https://busco.ezlab.org/). The clean reads of each sample were compared with the reference sequences obtained by Trinity assembly to obtain the mapping results of each sample. All transcripts obtained by this transcriptome sequencing were annotated against six databases (NR, Swiss-Prot, Pfam, COG, GO and KEGG databases), and the annotation situation in each database was statistically analyzed. The expression level of the transcript was quantitatively analyzed by RSEM software (http://deweylab.biostat.wisc.edu/rsem/). FPKM (Fragments Per Kilobases per Millionreads) was used to analyze the expression levels of differential genes. DESeq2 algorithms was used to select a subset of differentially expressed genes (DEGs) (adjusted *p* ≤ 0.05 and |log2 (fold-change) | ≥1). DEGs were considered as the targets for further analyses.

### qRT-PCR analysis

The expression levels of the selected genes in muscle tissues were determined by real-time PCR. The Primer 6.0 software was used to design primers (Table 3). Total RNA was isolated from the muscle with an animal tissue total RNA extraction kit (Fuji, Chengdu, China). cDNA was synthesized from 2 µg of RNA by a RT Easy™ II kit (Fuji). qPCR was performed by a SYBR green real-time PCR kit (Takara, Kyoto, Japan) and a Thermo Cycler (BioRad, Hercules, CA, USA). Ct values from Siberian sturgeon genes expression were normalized to Ct levels of *β-actin*, and the relative expression of genes was estimated by the 2^–ΔΔCT^ method.

**Table 3:** Primers used for qRT-PCR in this study

Gene	Sequence name	Sequence (5′-3′)	Tm (℃)
*mTORC1*	*TRINITY_DN12456_c0_g1*	F: CCCGAGCCACGCTCCATATTTC	59.8
		R: CGGCTGAAGCTTACAGCAGGCA	
*4E-BP1*	*TRINITY_DN4703_c0_g1*	F: CGGGAGGAACCCTGTTTAGTACCA	61.7
		R: CCTGGAATGTTGGGAAGGTAGCG	
*MYH9*	*TRINITY_DN8077_c0_g1*	F: CCTGCTCTTTCGTCTGTGCTTTCT	59.9
		R: TGGCTATGCTGAAGGTGGTGTCT	
*SOD1*	*TRINITY_DN5659_c0_g1*	F: AAGGAGGCTGGACCAGTGAAGTT	61.2
		R: TCATCTTGCGGCGCACCATG	
*UQCRFS1*	*TRINITY_DN722_c0_g1*	F: GGATAGCAGTGACGGTAGGAAGG	63.5
		R: ACAGTCTTGGCAGCGTAGGC	
*NDUFAB1*	*TRINITY_DN724_c0_g2*	F: CGTGTCCTGTATGTCCTGAAACTG	61.7
		R: TCAACCTGGTCCAAGCTGTCC	
*COX4*	*TRINITY_DN522_c0_g1*	F: TCGGTTGTTGCTGGAGTGTTCTTC	63.3
		R: CTCTGGGTCTGGGCAGCTATCC	
*COX6A*	*TRINITY_DN5536_c0_g1*	F: GCAGCAGGGCGACTCTTACAAC	62.5
		R: AGAGCCACCACGAAGGACAGG	
*ATPeF1A*	*TRINITY_DN5067_c0_g1*	F: AGAACTGGTGCTATTGTGGATGTG	61.7
		R: GCCAACTCTTCTACGCTCCTTAG	
*ATPeF1D*	*TRINITY_DN1360_c0_g1*	F: ACGGTGAACGCAGACTCCTC	63.5
		R: CGGACTGTGCTTTCTCCAGATTAG	
*SLC25A4S*	*TRINITY_DN2071_c0_g1*	F: CATCATACCCTTGGCAGTGTCGTAG	62.5
		R: GACCTCGCTGTGCTTCGTGTATC	
*β-actin*	*Reference gene*	F: TGAGGTAGTCAGTCAGGTCA	62.5
		R: TGGTCGTACCACTGGTATTG	

### Statistical analyses

All data were presented as mean ± SEM (*n* = 3). Significant difference was determined by using the one-way ANOVA in SPSS version 26.0 software, and histograms were drawn by GraphPad Prism 8. The *p* < 0.05 was considered statistically significant.

## Results

### Resveratrol promotes muscle fiber thickening

In order to explore whether resveratrol has an effect on the weight gain of Siberian sturgeon, fish were weighed before and after feeding on resveratrol. However, no significant differences were observed in final body weight (FBW) and percentage weight gain (PWG) between two groups (Fig. 1B).

The histological changes of muscle tissue were observed by H&E staining. As shown in Fig. 1C, the muscle fibers were more closely arranged and the muscle cell volume were increased after feeding on resveratrol. Besides, the diameter of muscle fibers was measured by Nikon TS100 light microscope, and it was found that the diameter of muscle fibers in R group was increased significantly (*p* < 0.01) (Fig. 1D).

Moreover, the expression levels of *mTORC1*, *4E-BP1* and *MYH9* in the muscle were measured (Fig. 1E), of which the expression levels of *mTORC1* were significantly increased.

### Resveratrol improves the nutritional value of Siberian sturgeon

To investigate the effect of resveratrol on the nutritional value of muscle of Siberian sturgeon, the amino acid content in muscle was detected (Table 4). As shown in Table 4, the content of serine and histidine in muscle of Siberian sturgeon was increased significantly after feeding on resveratrol. In addition, the content of the other 14 amino acids was increased, but not to a significant level. These results indicate that feeding a certain dose of resveratrol can improve the nutritional value of Siberian sturgeon.

**Table 4:** The composition and content of amino acids of muscle in Siberian sturgeon

	Dietary Resveratrol Levels, mg/kg		SEM	*p* values
	0	0.16		
Asp	1.71	1.92	0.08	0.21
Thr	0.76	0.85	0.03	0.18
Ser	0.67	0.75	0.03	0.03*
Glu	2.44	2.69	0.10	0.27
Gly	0.92	0.95	0.01	0.16
Ala	1.00	1.09	0.03	0.22
Val	0.83	0.92	0.03	0.23
Met	0.51	0.57	0.02	0.17
Ile	0.78	0.87	0.03	0.25
Leu	1.35	1.51	0.06	0.22
Tyr	0.58	0.65	0.03	0.19
Phe	0.73	0.81	0.03	0.23
Lys	1.63	1.83	0.08	0.24
His	0.53	0.62	0.02	0.03*
Arg	1.04	1.13	0.03	0.22
Pro	0.50	0.54	0.01	0.08
total amino acids	15.97	17.7	0.63	0.20

*p < 0.05

### Resveratrol enhances the activity of the antioxidative enzymes in the muscle

**Fig. 2:**
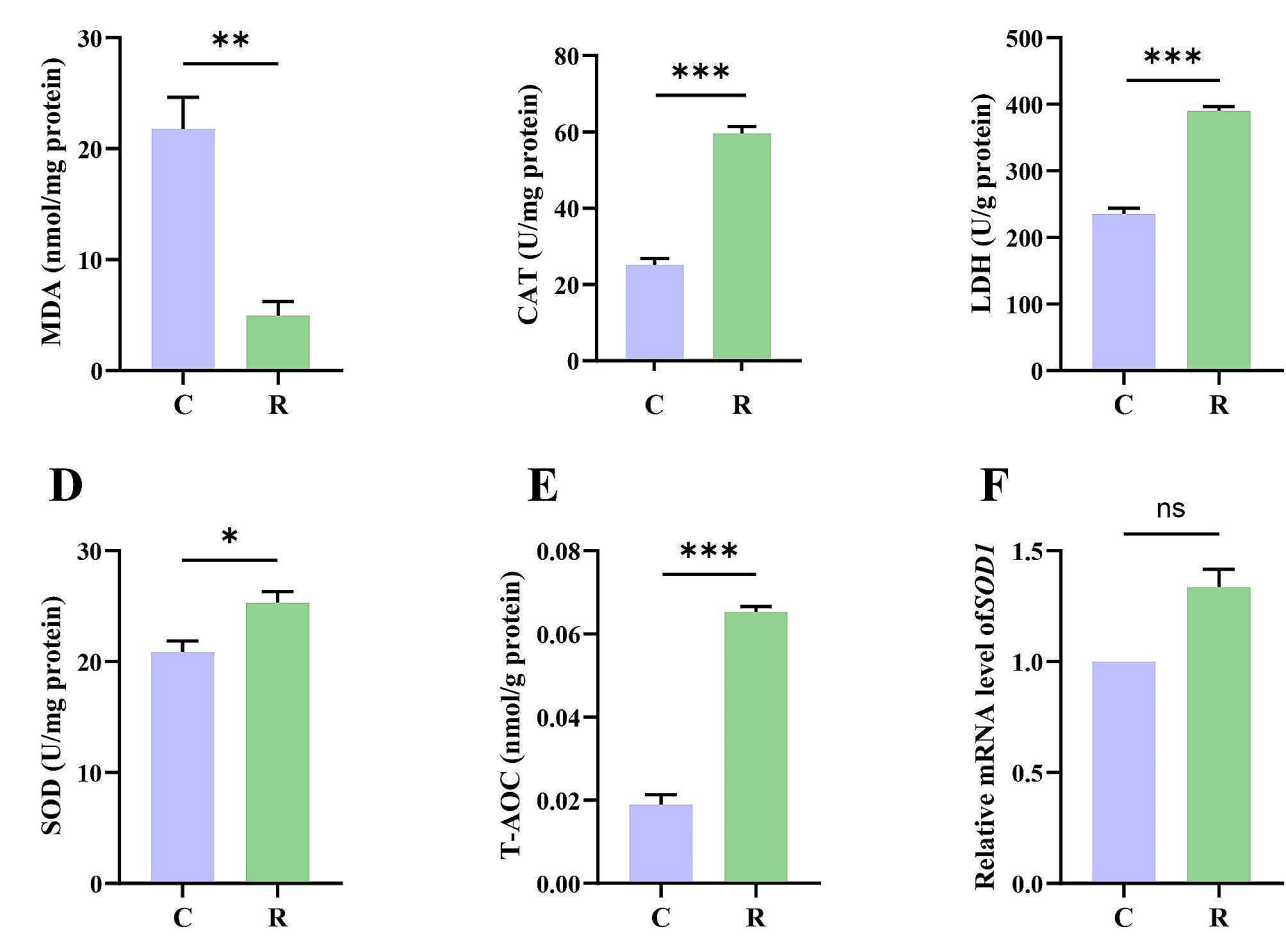
Effect of resveratrol on antioxidant system in the muscle of Siberian sturgeon. (**A**) The concentration of MDA. **(B, C, D)** CAT, LDH and SOD activity in the muscle. (**E**) T-AOC in the muscle. (**F**) The expression level of *SOD1* in muscle of Siberian sturgeon after feeding on resveratrol. Data were shown as mean ± SEM. *p < 0.05, **p < 0.01, ***p < 0.001. ns: no significance

To explore the effects of resveratrol on the antioxidative ability of muscle in Siberian sturgeon, the activity of the antioxidative enzymes was detected in muscle tissue of sturgeon after feeding on resveratrol (Fig. 2). The high increase of CAT, LDH, SOD and T-AOC activities was occurred in the muscle, indicating that the antioxidant capacity was enhanced after intaking resveratrol. Besides, the significant decrease of MDA level was observed.

### Resveratrol inhibits the ROS generation-related pathways

The transcriptome analysis of 6 samples was completed by Illumina Novaseq 6000, and the Clean Data of all samples reached more than 6.7 Gb, while the percentage of Q30 bases was more than 93.24%. A total of 59,910 unigenes and 92,003 transcripts expressed in this analysis were detected. A total of 2603 DEGs were obtained between the two groups, including 1373 up-regulated genes and 1230 down-regulated genes (Table 5). All genes and transcripts obtained by transcriptomic assembly were annotated against the six databases (NR, Swiss-Prot, Pfam, eggNOG, GO and KEGG), and the annotations in each database were shown in Table 6.

**Table 5:** The numbers of DEGs after feeding 0.16 mg/kg resveratrol

Group	Total DEGs	Up-regulated	Down-regulated
R vs.C	2603	1373	1230

**Table 6:** Annotation summary of Siberian Sturgeon muscle tissue transcriptome

Database-Annotated	Number of annotated unigenes	Percentage of annotated unigenes (%)
Annotated in GO	18,854	31.02
Annotated in KEGG	19,885	32.72
Annotated in eggNOG	22,828	37.56
Annotated in NR	27,794	45.73
Annotated in Swiss-Prot	21,244	34.96
Annotated in Pfam	18,222	29.98
Total annotated	28,217	46.43

**Fig. 3:**
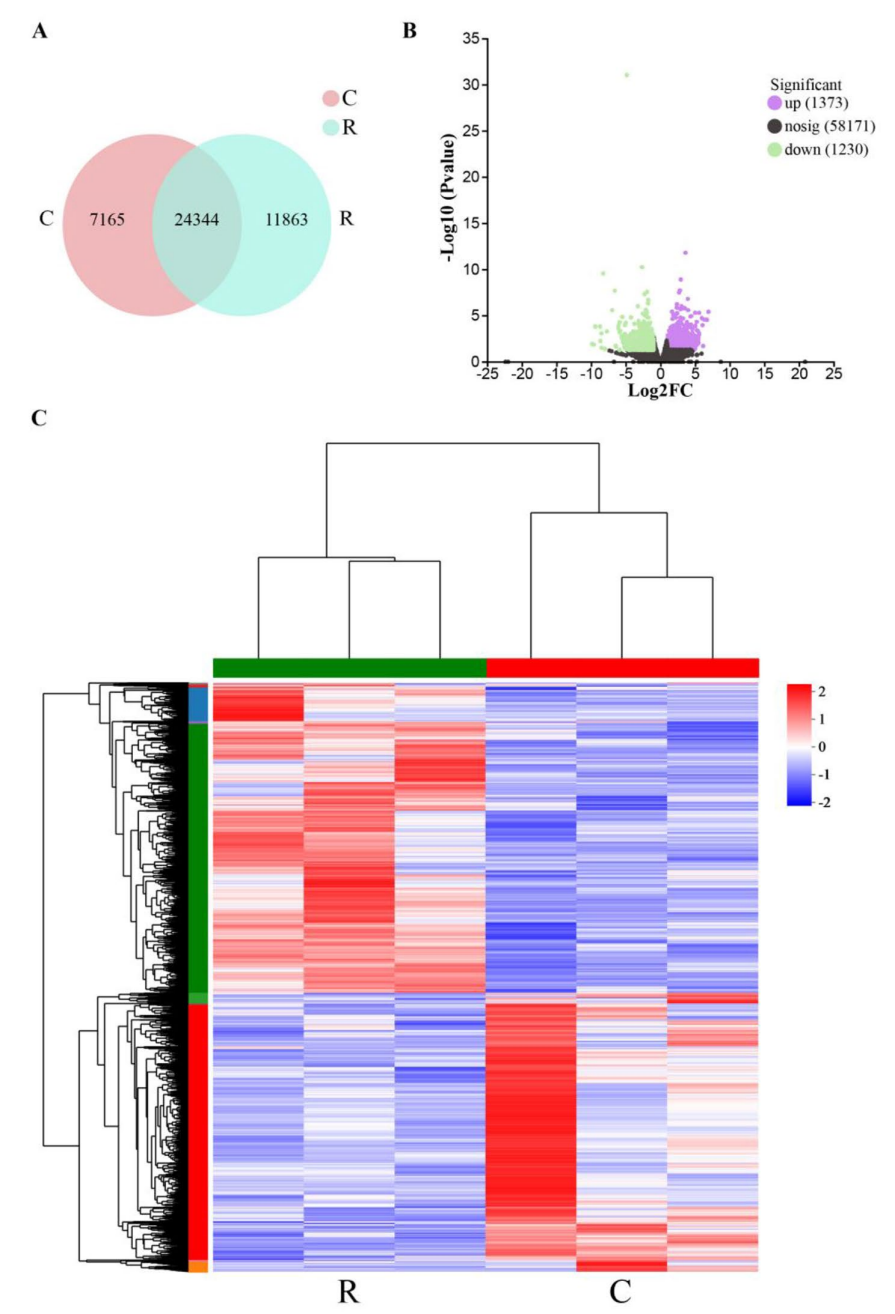
Effects of resveratrol on the transcriptomic dynamic changes of Siberian Sturgeon muscle. (**A**) Venn diagram of DEGs among C and R groups. (**B**) Volcano plot of DEGs in two groups. (**C**) Hierarchical clustering analysis based on FPKM of DEGs. Red and blue indicated that the gene expression level was up-regulated and down-regulated, respectively

Prior to DEGs analysis, the principal component and the intersample venn analysis were performed based on the expression matrix. From the venn plots, there were 11,863 unique genes in the R group, and 7165 unique genes in the C group, and 24,344 common genes expressed in both groups (Fig. 3A). Volcano plot (Fig. 3B) and hierarchical clustering analysis (Fig. 3C) showed that the DEGs distributed in the two groups, indicating that there are significant differences in gene expression.

**Fig. 4:**
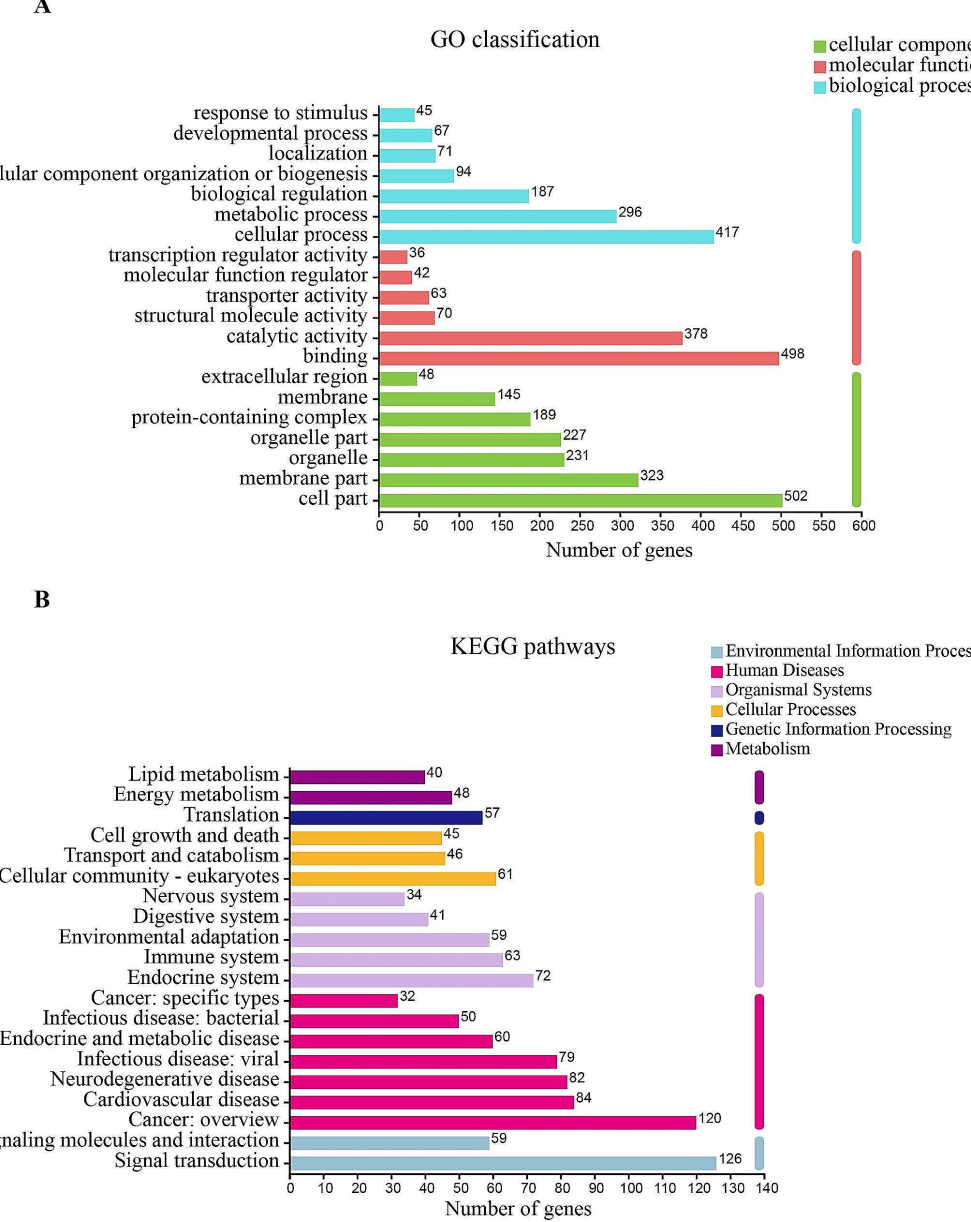
Effects of resveratrol on the transcriptomic dynamic changes of Siberian Sturgeon muscle and DEGs classification. (**A**) Histogram of GO annotation analysis. (**B**) Histogram of KEGG annotation analysis

The DEGs were then annotated by GO analysis, and the results showed that unigenes were classified into 3 GO term types, of which “Cell part”, “Cellular process”, and “Binding” were dominant in the categories “Cellular component”,“Biological process”, and “Molecular function”, respectively (Fig. 4A). To investigate the potential pathways involved in antioxidant function of muscle tissue in Siberian sturgeon fed with resveratrol, 2603 DEGs were classified into KEGG pathways, of which the pathway with most annotated unigenes was “Signal transduction” (126 unigenes), followed by “Cancer: overview” (120 unigenes), “Cardiovascular disease” (84 unigenes) and “Neurodegenerative disease” (82 unigenes) (Fig. 4B).

**Fig. 5:**
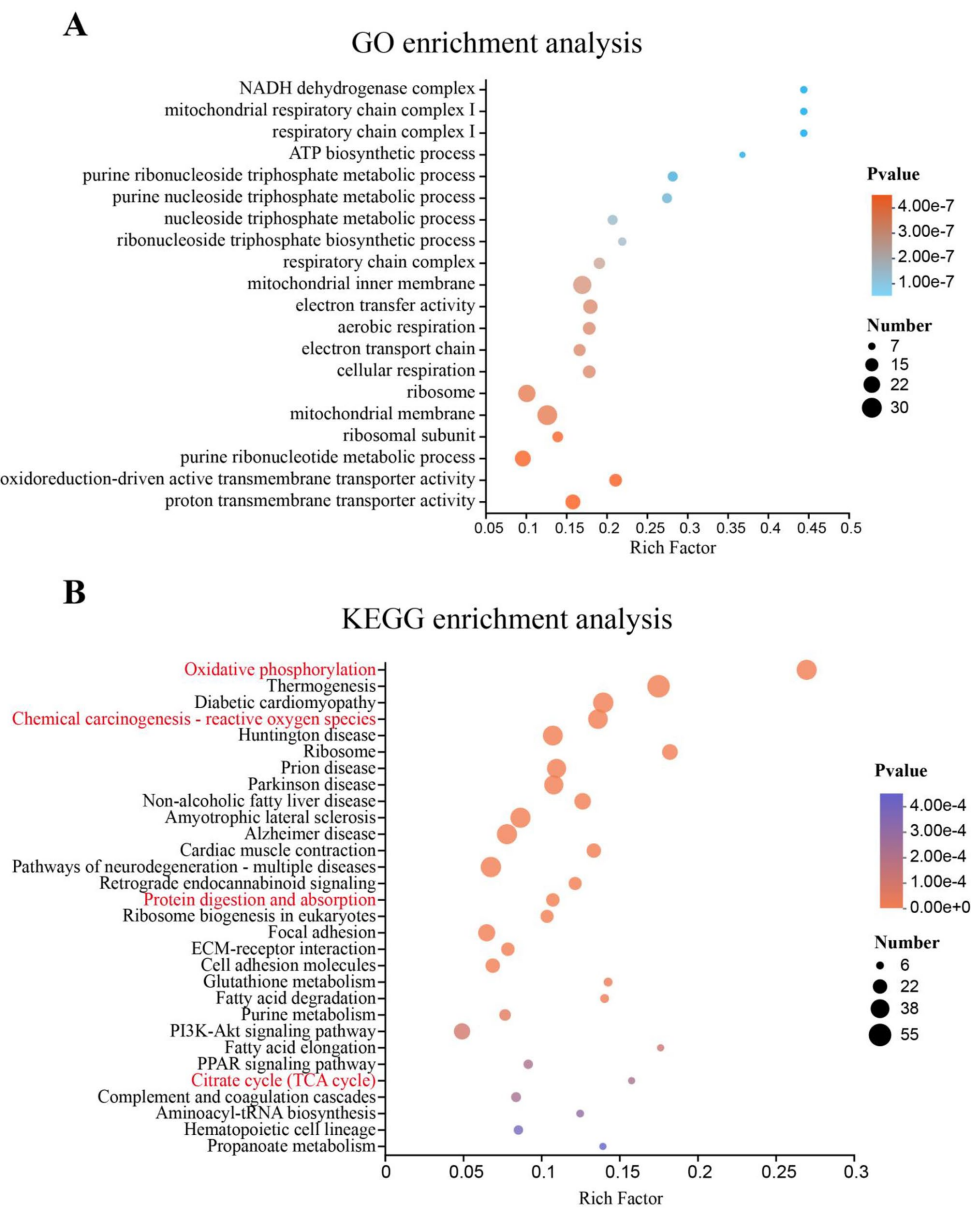
Enrichment analysis of muscle tissue transcriptome in Siberian sturgeon. (**A**) GO enrichment analysis. (**B**) KEGG enrichment analysis

To further investigate the DEGs involved in the antioxidant reaction between two groups, 509 genes were selected for GO enrichment analysis based on KEGG annotation. The results showed that the top 20 significant enrichment GO terms were mostly related to the electron respiratory transport chain process in mitochondria (Fig. 5A). Moreover, 455 genes were selected for KEGG enrichment analysis. Notably, in the top 30 significant enrichment pathways, the antioxidant-related aspects such as “Oxidative phosphorylation” and “Chemical carcinogenesis - reactive oxygen species” were the main enrichment pathways (Fig. 5B).

**Fig. 6:**
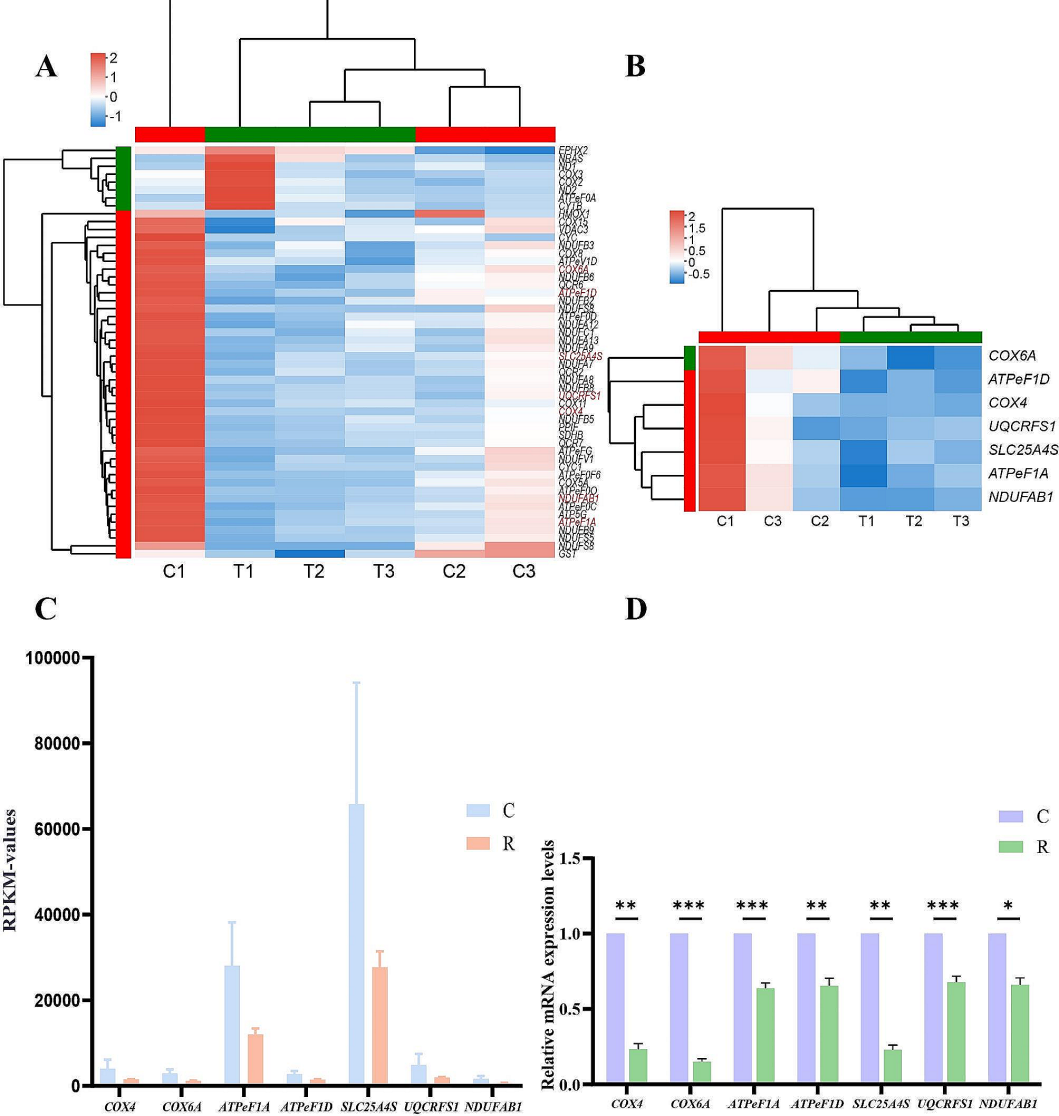
Heatmap of the oxidative phosphorylation-related DEGs, comparison of the expression of 7 selected DEGs by RNA-seq and qRT-PCR. (**A**) Heatmap of oxidative phosphorylation and ROS generation-related DEGs. (**B**) Heatmap of 7 selected DEGs. (**C, D**) Comparison of the expression of 7 selected DEGs by RNA-seq and qRT-PCR. The qPCR results were calculated by normalizing to the reference gene (*β-actin*), Mean ± SEM. *p < 0.05, **p < 0.01, ***p < 0.001

Furthermore, cluster of 46 DEGs enriched in “Oxidative phosphorylation” and “Chemical carcinogenesis - reactive oxygen species” pathways was analyzed (Fig. 6A). 7 DEGs related to Oxidative phosphorylation and ROS generation were selected for cluster analysis, the result showed that there were significantly different expression patterns between C group and R group (Fig. 6B). To verify the validity and accuracy of transcriptome data, 7 DEGs were selected from the antioxidant-related pathways (“Oxidative phosphorylation” and “Chemical carcinogenesis-reactive oxygen species”) for qRT-PCR. As shown in Fig. 5D, the relative gene expression change trend obtained by transcriptome sequencing and qRT-PCR was consistent, indicating the accuracy and reliability of transcriptome data. The expression levels of genes (*UQCRFS1, NDUFAB1, COX4, COX6A, ATPeF1A, ATPeF1D*) associated with mitochondrial complex were significantly down-regulated, implying that the electron transport chain on the mitochondrial membrane is inhibited, which results in the inhibition of ROS generation. Meanwhile, compared to C group, the expression level of *SLC25A4S* mediating the transmission of ROS was significantly decreased in R group, indicating that ROS is strongly induced in the muscle of Siberian Sturgeon fed with resveratrol.

## Discussion

In aquaculture, sturgeons tend to live in confined spaces with restricted movement which can damage their flesh quality [30–33]. Many studies have shown that resveratrol can ameliorate the flesh quality of livestock and poultry [30, 34, 35]. However, the effect of resveratrol on flesh quality of Siberian sturgeon has not been reported. In order to investigate the effects of resveratrol on flesh quality of Siberian sturgeon, the antioxidant capacity, tissue structure, nutritional composition and transcriptome of muscle of sturgeon were observed after feeding with resveratrol.

The muscular hardness, affected by myofiber diameter, is one of the important indexes of flesh quality [36, 37]. The skeletal muscle myogenesis is dependent on the ability of myoblasts to proliferate, synthesize proteins and fuse into myotubes [38, 39], which further impacts on the myofiber diameter. In our study, we observed that myofiber thickened after feeding on resveratrol (Fig. 1C), which was consistent with Rondinelle’s research results on Pacu fish [40]. Further research found, as shown in the 15th enrichment pathway in KEGG enrichment analysis (Fig. 5B), genes related to protein digestion and absorption were significantly enriched in current study. Meanwhile, after feeding on resveratrol, the amino acid content in muscle presented an increasing trend (Table 4). Combining our previous research [7], we speculated that this trend was attributed to the fact that resveratrol could promote the digestion and absorption of protein and amino acid, which are abundant in fish feed. Not only was the raw materials for protein synthesis in the muscle of Siberian sturgeon increased, but also the intracellular amino acids activated mTORC1 promoting protein synthesis by phosphorylating eukaryotic translation initiation factor binding protein (4E-BPs) [41, 42]. In this study, the expression level of *mTORC1* was significantly up-regulated and the expression level of *4E-BP1* was significantly down-regulated (Fig. 1E), indicating that resveratrol may stimulate protein production through mTORC1/4E-BP1 pathway, which promoted muscle myogenesis, thickened the diameter of muscle fibers and ultimately led to increased the muscular hardness. In addition, Actin is one of muscle structural proteins. MYH9 is involved in the regulation and tight junction of actin skeleton [43]. The increase of its gene expression (Fig. 1E) indicates that resveratrol can promote muscle growth and development.

Another important aspect of flesh quality is oxidative processes, which impacts on the meat quality characteristics (meat color, tenderness, texture, water-holding capacity, etc.), also called spoilage [44]. The higher the ROS content is in muscle, the easier it is to oxidize proteins and lipids, resulting in meat quality deterioration [45]. In muscle tissue, ROS is mainly cleared by antioxidant enzymes [46, 47]. In order to evaluate the effects of the dietary resveratrol on the antioxidant capacity of muscle of Siberian sturgeon, we detected MDA content, LDH, CAT, SOD activity and T-AOC of muscle after feeding on resveratrol (Fig. 2). MDA is a good biomarker of protein oxidation and lipid peroxidation in animal tissues [48]. In the present study, compared with the control group, MDA level in muscle of Siberian sturgeon was decreased significantly, suggesting that resveratrol depresses the oxidation of proteins and lipids in the muscle. SOD and CAT play significant roles in scavenging ROS– superoxide free radical (•O^2−^) and hydrogen peroxide (H_2_O_2_), respectively [48, 49]. The enhancement of SOD and CAT activity can reflect the decrease of •O^2−^ and H_2_O_2_ content in muscle. LDH catalyzes the conversion of pyruvate to lactic acid in the cytosol, accompanied by the conversion of NADH to NAD^+^, which reduces the raw material of the electron transport chain and reduces the production of ROS at the source [50]. The enhancement of LDH activity indicates that the oxidative stress of muscle fibers is delayed. In short, the enhancement of antioxidant enzyme activity reflected the improvement of the antioxidant capacity of muscle. We speculated that this change would slow down the deterioration of flesh and improve the quality of meat. These results were similar to the previous study, which found that resveratrol enhanced CAT activity and total antioxidative capacity (T-AOC) in muscle of broilers [51].

With the purpose of exploring the internal mechanism of resveratrol strengthening the antioxidant capacity of muscle, transcriptome analysis was performed on muscle tissue. KEGG enrichment analysis revealed that “Oxidative phosphorylation” and “Chemical carcinogenesis - reactive oxygen species” were enriched to the top 10 pathways (Fig. 5B). We selected 7 key genes in these two pathways (*UQCRFS1, NDUFAB1, COX4, COX6A, ATPeF1A, ATPeF1D, SOD-1*) for gene expression verification (Fig. 6D). NDUFAB1 is the subunit of NADH dehydrogenase, and NADH is the electron donor of the mitochondrial electron transport chain [52]. In this study, the significant down-regulation of *NDUFAB1* indicates that the starting point of the electron transport chain is inhibited, and the number of hydrogen ions and electron transport in mitochondria is reduced. Respectively, UQCRFS1, COX4 and COX6A are subunits of electron transport chain complex III and complex IV, which are considerable sites for producing ROS [53, 54]. The expression levels of *UQCRFS1, COX4* and *COX6A* were significantly decreased, indicating that the process of electron transport is inhibited. We speculated that this would lead to a decrease in the probability of oxygen and electron contact on the mitochondrial inner membrane, which implies that less ROS will be generated. ATPeF1A and ATPeF1D are subunits of ATP synthase [55], and the decrease of their gene expression indicates the reduction of ATP production in mitochondria. We speculated that this was attributed to the fact that resveratrol enhances the activity of muscle LDH enzyme (Fig. 2C), and previous study have shown that the enhanced activity of LDH enzyme can make mitochondria produce low demand for ATP through oxidative phosphorylation, avoiding the increase of ROS in mitochondria [50]. SLC25A4S, one of mitochondrial permeability transition pore (mPTP), plays an important role in a pathway for •O^2−^ to exit the mitochondria [56]. We found that the gene expression of *SLC25A4S* was down-regulated significantly, indicating less •O^2−^ is transported into the cytoplasm. SOD1 is a typical type of SOD family in antioxidant defense system. The increase of gene expression of SOD1 indicates the enhancement of enzyme activity, which suggests that the active oxygen scavenging ability of muscle fibers is promoted [57]. Our results are consistent with the earlier report that the antioxidative stress capability of resveratrol was confirmed by increasing the gene expression of *SOD1*, and SOD enzyme activity and decreasing ROS activity [58]. Taken together, these results suggest that resveratrol can improve the antioxidant capacity of muscle by reducing the production of ROS and enhancing the ability to remove ROS in muscle fibers.

## Conclusion

In summary, our study reveals that resveratrol enhances the flesh quality of Siberian sturgeon by thickening muscle fiber diameter, and increasing the nutritional value and the antioxidant capacity of muscle. This study will contribute to understand the mechanism of antioxidants improving flesh quality of fish, and promote the development of aquaculture.

## Data Availability

No datasets were generated or analysed during the current study.

## Abbreviations

qRT-PCR: Quantitative real-time PCR
GO: Gene Ontology
KEGG: Kyoto Encyclopedia of Genes and Genomes
